# Optical wireless communication

**DOI:** 10.1098/rsta.2020.0051

**Published:** 2020-03-02

**Authors:** Harald Haas, Jaafar Elmirghani, Ian White

**Affiliations:** 1 School of Engineering, LiFi Research and Development Centre, Institute for Digital Communications, University of Edinburgh, Edinburgh EH9 3JL, UK; 2 School of Electronic and Electrical Engineering, Institute of Communication and Power Networks, University of Leeds, Leeds LS2 9JT, UK; 3 Vice-Chancellor's Office, University of Bath, Claverton Down, Bath BA2 7AY, UK

**Keywords:** optical wireless, LiFi, visible light communication, free space, attocell

## Abstract

Optical wireless communication has attracted significant interest recently in industry and academia. This special issue features a collection of inter-related papers with the intention to cover all necessary multidisciplinary challenges to realize optical wireless networks. We hope that this special issue will serve as a comprehensive reference and that it will be a resource which fosters many more new ideas for this rapidly emerging field.

This article is part of the theme issue ‘Optical wireless communication’.

## 1. Setting the scene

Wireless communication has undoubtedly become an essential utility of our every-day lives. Most of the existing wireless communication systems use radio frequency (RF) technologies to convey information. However, with an ever-growing demand for wireless data, fuelled by new paradigms such as machine-type communication for autonomous systems as well as new devices such as intelligent glasses using augmented reality and virtual reality (VR), there is general agreement that the RF spectrum will not be sufficient to future-proof wireless communication. Consequently, it is imperative to consider also the optical spectrum for wireless communication.

The optical spectrum already is a key enabler for the global Internet. Optical fibre communication networks not only connect all continents, they also form the backbone of modern communication networks that provide high-speed data access to metropoles, cities, towns and increasingly also to homes. Extending the optical fibre medium to include the free-space medium for last mile connectivity and mobile access not only seems a natural step, but also one that is relatively straightforward to take.

Therefore, in recent years, there has been a significantly increasing interest from academia and industry in optical wireless communication (OWC) technologies. There is also now a wide range of OWC technologies, as a result of the many use cases they can serve. The main OWC technologies are (i) free space optical (FSO) communications, (ii) visible light communications (VLC), (iii) optical camera communications (OCC) and (iv) wireless networking with light, which is also referred to as LiFi.

FSO communication is the closest functionally to optical fibre communications as it provides static wireless point-to-point communications over relatively large distances of up to tens of kilometres. FSO systems primarily use the infrared spectrum. With the advent of the blue light emitting diode (LED) which led to the development of the bright white LED, visible light communication (VLC) emerged. The first VLC application which used white LEDs was for wireless audio signal transmission [1]. VLC was primarily pioneered by Prof. Nakagawa and co-workers at Keio University [2] at the beginning of the millennium.

In a similar way to how white high brightness LEDs have led to VLC, the digital camera in smartphones has led to OCCs, because it is possible to use this sensor to detect information encoded in flickering light [3,4]. We have therefore included a review article of OCC techniques in this special issue by Liu & Xu [5].

**Figure 1.**
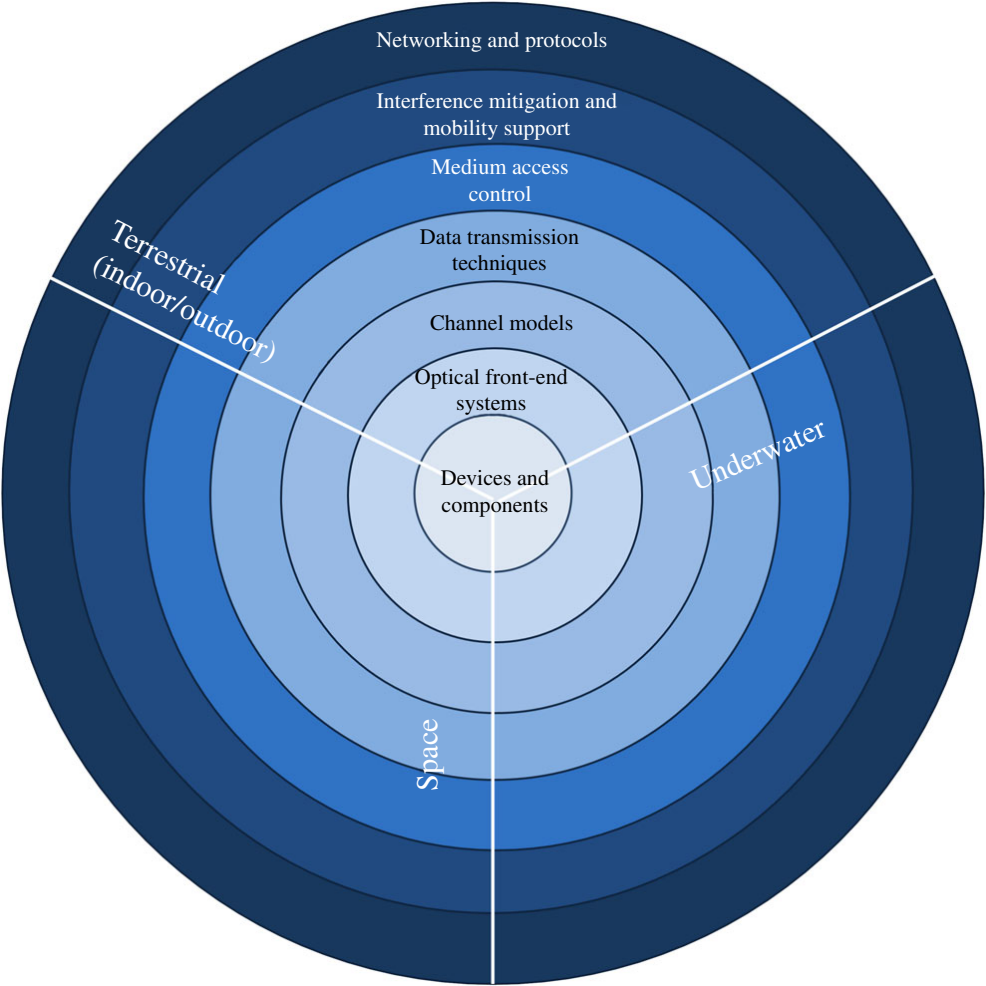
Basic building block technologies required to build OWC networks for possible terrestrial, underwater and space deployments. (Online version in colour.)

Lastly, and this is the main focus of this theme issue, the connection of a random number of mobile terminals to an infrastructure that is composed of multiple fixed optical access points each covering a small area defines wireless networking with light [6]. The communication links are expected to be bi-directional. Furthermore, the connection is expected to be seamlessly maintained when mobile terminals roam between optical access points. Wireless networking using light requires a set of basic technologies, which are depicted in figure 1. The principal technologies at the outer layers, starting with channel models, are the same as in RF networks. However, due to the properties of light, unique solutions are required for each category. New solutions are essentially dependent on the actual channel models, the optical front-end systems and the devices and components.

Therefore, optical devices and components are most critical as they determine key system parameters such as peak data rates, link distance, etc. The optical front-end systems are equally important as they have a significant impact on the actual link budget, which is constrained by the maximum optical output power and the receiver sensitivity. The link budget seamlessly feeds into the channel models, which vary largely with the actual deployment scenario. The data transmission techniques aim to maximize the data rate subject to the actual communication channel. Since multiple mobile terminals will need to be supported simultaneously, there is contention for the available channel capacity. Therefore, medium access control (MAC) protocols need to be developed to ensure fairness and to guarantee quality of service. Because there are multiple optical access points in a network, it is unavoidable that light cones overlap, which can lead to interference. This is particularly severe at the edges of a light cone. Users at these locations suffer from low signal to interference plus noise ratios (SINR) which severely limits data rates. Techniques will need to be developed which mitigate interference and ensure that the SINR is sufficiently high at any orientation and location. Lastly, the optical wireless networks will need to be integrated in existing RF heterogeneous wireless networks using principles such as software-defined networking (SDN).

Figure 1 also shows the basic application areas. We note that the optical spectrum can also be used to build wireless networks underwater, which is difficult to achieve with the RF spectrum. However, the initial application area will be terrestrial indoors since about 80–90% of all Internet traffic originates and terminates indoors. This would also provide an opportunity to harness the lighting infrastructure to build optical wireless networks on top of it. The downlink direction, therefore, could be based on white light while the uplink uses the infrared spectrum. This conveniently allows for full duplex operation, which for delay-sensitive applications is very advantageous.

This theme issue features leading-edge scientific contributions in the outlined key areas toward the development of OWC networks. It contains a balanced mix of invited review papers and invited original research papers by internationally renowned experts in the field. The state of the art of the field is provided for all the fundamental building blocks illustrated in figure 1. Furthermore, this theme issue offers wide-ranging insights into where this technology will be taken in the future.

## 2. Challenges in optical wireless communication networking

In the following, we will briefly introduce some of the key challenges in all the different research areas.

### (a) Devices and components

**Figure 2.**
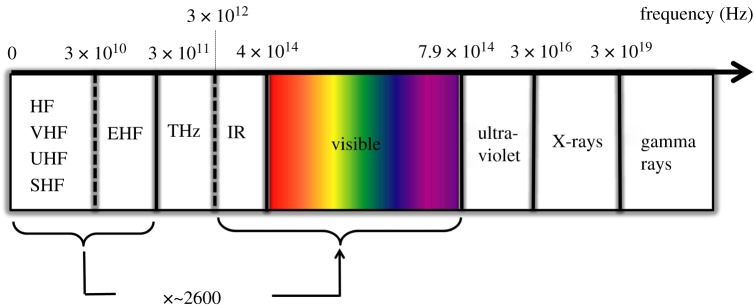
The entire electromagnetic spectrum. The combined infrared and visible light spectra are 2600 times larger than the entire RF spectrum. (Online version in colour.)

While the optical spectrum is three orders of magnitude larger than the entire RF spectrum (figure 2), there are fundamental limitations that currently prevent us from being able to fully harnesses this huge amount of wireless transmission resource—especially when using LEDs as transmitters.

**Figure 3.**
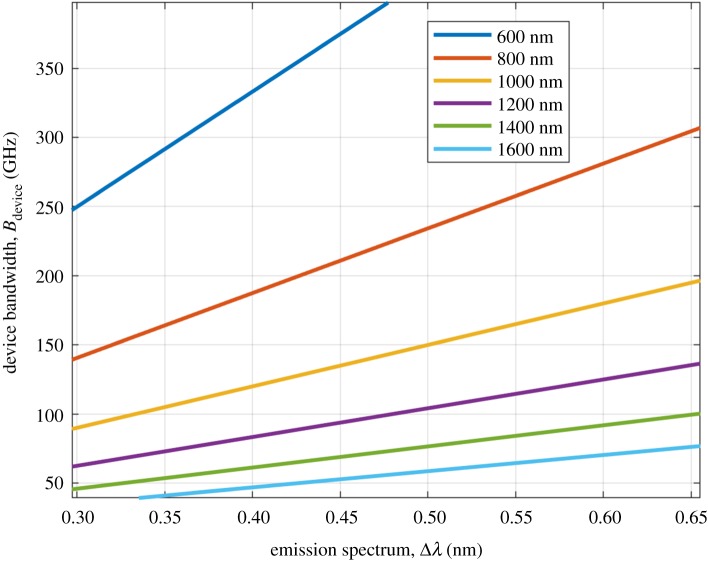
Required device bandwidth as function of emission spectrum. (Online version in colour.)

This is because the bandwidth of typical LED devices is limited to 10 s and 100 s of MHz [7]. This limits the data speeds of single LEDs, typically to below 10 Gbps [8]. However, to overcome this limitation, it is possible to use multiple devices of similar bandwidth, but at a different emission spectrum, which is referred to as wavelength division multiplexing (WDM). In [9], it was shown that four standard off-the-shelf LEDs have yielded an aggregate data rate of 15.7 Gbps despite the partial overlap of their emission spectra. An optimum use of WDM would require sub-nanometre spectral emission masks and high device bandwidth. The required device bandwidth can be approximated as follows:

2.1 $$B_{\text{device}}\approx c\frac{\Delta \lambda }{\lambda^{2}},$$

where *c* is the speed of light in vacuum, Δ*λ* is the emission spectrum of the light source assuming a rectangular spectrum mask, and *λ* is the centre wavelength. Equation (2.1) is plotted in figure 3 as a function of the centre wavelength of the emission spectrum.

From figure 3, it can be seen that a red light source (600 nm) with an emission spectrum of 0.45 nm would require a device bandwidth in excess of 350 GHz.^1^ First, it is clearly not possible to achieve such a narrow emission spectrum with current LEDs, and second, it is practically difficult to achieve a transmit device bandwidth of 350 GHz with current technology [10], let alone achieving detectors that have a bandwidth of 350 GHz. Overcoming the device limitations is an area of active research. Unlike point-to-point communication, such as in FSO, wireless networking systems have to provide simultaneous wireless connections to a potentially very large number of end users in indoor environments. There is, therefore, an additional challenge (in addition to device bandwidth) which is related to link margins and receiver sensitivity due to the small detector size on the one hand, and the widely spread optical power on the other hand. In the short- to medium-term, laser-based lighting may offer the next step towards higher device bandwidths while providing high optical output power to enhance the link data rate performance and maintaining eye-safety requirements. In addition, there is a wealth of research papers on the use of micro LEDs for high-speed OWCs. The major limitation of these devices is the low optical output power, but arrays of micro LEDs can be used to overcome this limitation. This issue features a review article by Dawson *et al.*, who have been pioneering the development of micro LEDs for high-speed VLC [11]. Various laser devices such as vertical-cavity surface-emitting lasers (VCSELs) operating in the infrared spectrum have also been used to build high-speed OWC links. This issue features a research article by Koonen *et al*., who have been leading beam-steering laser-based optical multiuser systems, which achieved tens of gigabit per second transmission speeds [12].

Although inorganic semi-conductor devices have primarily been used as optical transmitter and receiver devices, organic semiconductors exhibit properties which make them ideal candidates for optical devices in VLC systems. In particular, their flexibility can be used to bend them around edges which can be helpful to increase connectivity when the device is randomly oriented. While it has been shown that it is possible to achieve gigabit per second links using organic LEDs (OLEDs) [13], it has also been demonstrated that organic photovoltaic cells can be used as high-speed data detectors. This issue includes a review article by Samuel and co-workers [14], who have been leading the development of record achieving organic VLC devices.

OWC systems primarily use intensity modulation/direct detection (IM/DD). The information is encoded in changes of the light intensity. For the decoding of the information, a square-law detector is used—typically, a photodiode (PD). The opto-electric conversion is described by means of the responsivity, which is defined as

2.2 $$R=\frac{\eta q\lambda }{hc}\approx \frac{\eta \lambda [\mu m]}{1.24},$$

where *η* is the quantum efficiency defined as the *carrier generation rate* divided by the *photon incident rate*, *q* is the elementary charge in Coulomb, and *h* is the Planck constant. Because the quantum efficiency is less than unity, the maximum responsivity at a wavelength of 600 nm is approximately 0.5 A/W, which poses a severe limitation on the receiver sensitivity. Therefore, avalanche photodetectors (APDs) have been considered. These devices operate with a very high electric field so that an electron that is generated from an incident photon gains very high kinetic energy and generates new electron-hole pairs. This process is called ionization. The newly generated electrons are also subjected to the same high electric field and are thus able to generate new electron-hole pairs, themselves creating an avalanche effect. APDs that are biased beyond the breakdown voltage are referred to as single-photon avalanche diodes (SPADs). SPADs operate in the Geiger mode and can detect single photons. This theme issue includes an article by Henderson and co-workers [15], who is a pioneer of digital SPAD devices in complementary metal-oxide semiconductor (CMOS).

### (b) Optical front-end systems

The optical front-end can be divided into the transmitter part and the receiver part. Because the objective is to build bi-directional communication links, both parts have to be integrated into a single transceiver unit. Sufficient decoupling of the received and transmitted signals is important and various duplexing techniques can be used. Trade-offs to consider in the design of the duplexing techniques are bandwidth, latency and data rate [16,17].

The optical system of the transmitter has to ensure that eye-safety standards are met [18] while achieving quality of service requirements. This can be challenging when the transmitter is a point source. It is, therefore, often desirable to convert the point source into an extended source which changes eye-safety conditions.

One of the key challenges in mobile networking is that the mobile terminal randomly changes orientation. Moreover, the link between the mobile terminal and the fixed access point can be obstructed. In a system that heavily relies on line-of-sight (LoS) link conditions, this becomes a critical challenge. A possible solution to this challenge is to provide link diversity by means of multiple simultaneously active transmitters whose position and possibly orientation is different. This leads to the concept of angular diversity transmitters [19,20].

At the receiver, the challenge is to capture enough photons to ensure correct detection of the transmitted information. Ideally, the receiver would have a large area to allow the system to collect a large number of photons. However, a large-area detector typically has a low bandwidth because the large area results in a detector that has a high capacitance. Therefore, often concentrators are used. The maximum concentration gain is governed by conservation of etendue [21]:

2.3 $$G_{\text{max}}=\frac{n^{2}}{\sin^{2}(\theta )},$$

where *n* is the refractive index of the concentrator material and *θ* is its half-angle. As seen from (2.3), if a large gain is desired, the half-angle has to be small. Apart from this limitation, there is also a limitation with respect to the height of a concentrator, which typically is tens of millimetres which makes it difficult to integrate such a device into a mobile phone. The ideal receiver optical sub-system (i) is flat, (ii) uses small detectors and (iii) allows for a half-angle close to 90° to avoid strict alignment. Recently, there has been work on optical concentrators which is a candidate technology to overcome these challenges [22]. The same group has provided a review article on the design of optical front-end design for high-speed OWCs [23]. Moreover, there has been very interesting research on ultra-low impedance transimpedance amplifiers which lead to significant bandwidth enhancements [24]. This is because the dominating time constant which determines the device bandwidth is a product of capacitance and resistance. Ensuring a low resistance is therefore a promising direction to reduce the time constant and increase the bandwidth.

Similar to angular diversity transmitters, there has also been a lot of work on angular diversity receivers to overcome link obstruction and alignment issues [25–27].

### (c) Channel models

An important system element is the optical free-space propagation channel. The optimum design of the transceiver depends on the optical channel characteristics. Since objects act as reflectors, an impulse sent by the transmitter arrives at the receiver via multiple delayed paths. This is referred to as multipath propagation. Multipath propagation causes inter-symbol interference. This means that a transmitted symbol is corrupted by *n* previous symbols. This effect has to be eliminated by means of digital equalization, or has to be avoided by a proper selection of transmission techniques. Moreover, there may not always be a LoS channel. The receiver has to be able to cope with the situation that there are only reflections. It is, therefore, important to get a comprehensive understanding of (i) the power included in the reflected paths (in addition to the power received on the LoS path), and (ii) the temporal spread of the reflected signals. Furthermore, the reflectivity of the materials used indoors is wavelength-dependent. A full understanding of the wavelength dependent reflectance and absorption of objects and materials in typical deployment scenarios such as homes, offices and manufacturing plants is paramount to the development of OWC networks. In this issue, we have a contribution by Miramirkhani & Uysal [28], who have been at the forefront of the development of channel models for OWC systems. The same group has provided reference channel models to the IEEE 802.11bb standardization group. This standard is the first that defines wireless light-based networking systems.

### (d) Data transmission techniques

As stated above, the dominant data transmission mechanism is IM/DD. Since the optical signal is constrained to be strictly positive and real, the classic Shannonian theory is not applicable. The channel capacity is still unknown. However, Hranilovic *et al.* were the first to develop upper and lower information-theoretic bounds for such systems. The same group has provided a review article for this special issue [29]. Knowledge of the optimum signal distribution is invaluable for the design of optimum modulation techniques.

Although IM/DD is the major transmission technique used in OWC, it is possible to use coherent transmission at the cost of increased transceiver complexity. Recently, there has been an increasing number of works on coherent OWC systems. This is a direction that this field may also take in the future.

The most basic data transmission technique for IM/DD systems is on-off keying (OOK). OOK, however, severely limits the spectrum efficiency since in every transmission step or channel use only one bit is transmitted. Because the system is limited by the device bandwidth, this results in a hugely suboptimum use of the available bandwidth. However, OOK benefits from implementation simplicity and it is also robust to device nonlinearities. In addition, OOK severely suffers from inter-symbol interference when it is deployed at high data rates. It is desirable to use data transmission techniques that are more spectrum efficient, i.e. transmit multiple bits per channel use while increasing the robustness to multipath propagation. One such technique is orthogonal frequency division multiplexing (OFDM) which sends data symbols simultaneously on orthogonal subchannels. This is achieved by using the inverse fast Fourier transformation (IFFT) at the transmitter and the fast Fourier transformation (FFT) at the receiver. The parallel transmission means that the OFDM symbol is much longer than the maximum multipath delay. Therefore, a single-tap equalizer can be deployed. Also, every subchannel can carry a complex data symbol out of an alphabet of *M* total symbols where every symbol carries log_2_(*M*) bits. However, since the OFDM symbol is bi-polar, the signal needs to be subjected to a direct current (DC) bias before exposing the signal to the light transmitter, which operates solely in the first quadrant and thus cannot accept negative signals. This measure has a negative impact on the energy efficiency as the DC bias power is wasted unless it is used for other purposes such as illumination. However, while illumination might be desirable if an access point is combined with an illumination device, it is certainly undesirable in a mobile device such as a smartphone. Therefore, new digital modulation techniques have been developed based on multi-stream, or layered data transmission in conjunction with superposition modulation and iterative detection. This issue features a comprehensive review article on layered transmission techniques by Lowery [30], who has developed many novel digital modulation techniques for IM/DD systems.

OFDM is based on the Fourier transformation, which is an orthogonal transformation. However, there are other orthogonal transformation techniques such as the Walsh–Hadamard transform (WHT). This issue features a review article by Brand-Pearce *et al.* [31], who have pioneered this technique.

An alternative high spectrum efficiency achieving digital modulation technique for IM/DD is carrierless and amplitude phase modulation (CAP). CAP allows the transmission of high-order modulation symbols while maintaining the basic requirements of time domain IM/DD signals. This theme issue includes an overview article on CAP [32] by Bamiedaksis *et al.*, who have been leading the development and implementation of CAP techniques for optical communication systems.

Due to the bandwidth limitation of the devices, it is paramount to consider other dimensions for data encoding—in particular, the spatial dimension and the wavelength dimension. Multiple-input multiple-output (MIMO) techniques harness the spatial dimension. There are two fundamental techniques in OWC systems: (i) imaging MIMO and (ii) non-imaging MIMO. Imaging MIMO is very intolerant to movement and is thus more suitable for FSO-type systems while non-imaging MIMO can be used in mobile applications. However, it is usually not guaranteed that the MIMO channel matrix is always full rank, which is a necessity to fully benefit from maximum spatial multiplexing gains. In addition, MIMO systems suffer from inter-channel interference or cross-talk. Iterative interference cancellation techniques can be used to mitigate this effect, but these techniques require significant computational processing power. This theme issue includes a research article on networked MIMO techniques by Wang & Chen [33], who have significantly contributed to advancing the field of networked MIMO both theoretically and practically.

A MIMO technique which has attracted large interest in academia and industry is spatial modulation [34]. Spatial modulation completely avoids inter-channel interference and hence results in low computationally complexity MIMO decoding techniques. We have included a research article on spatial modulation for IM/DD systems [35] in this theme issue due to the practical relevance of this technique.

One of the unique properties of OWC is its capability to achieve high physical layer security due to the ability to easily contain light waves spatially. Lampe *et al.* [36] have led the analysis of the secrecy capacity of OWC systems. We are therefore pleased that this group has shared their latest research findings in this theme issue.

### (e) Medium access control protocols

In OWC wireless networks, the simultaneous support of many mobile terminals from a single access point (e.g. a light in the ceiling) is essential. These mobile terminals may have different service requirements. For example, in a manufacturing plant, the transmission delay may be more important than the peak data rate. It is, therefore, important to first develop optimum multiuser access techniques which avoid multiuser interference and achieve high spectrum efficiency. Ideally, the sum data rate of an access point is greater when supporting many users as compared to a single-user scenario. A candidate technology which can achieve this is non-orthogonal multiuser access [37]. This issue features the following articles which are related to multiuser access in OWC networks [31,38].

### (f) Interference mitigation and mobility support

A wireless network is characterized by many access points which are spatially distributed. Every access point covers a certain area. If a mobile device enters the coverage zone of an access point, the system performs a handover from the access point to which the mobile was previously connected to the new access point. This process needs to be fast enough to ensure uninterrupted wireless service provision. If the coverage of an access point is small, the requirement of a fast handover becomes particularly important. The key advantage of optical wireless networking is that very small cells can be generated where a cell is defined by the coverage area of an access point. This is a very important advantage because the same transmission resource can be reused many more times when compared to large area cells. This principle of cell shrinkage is the main reason in RF communication for the improvements in data rates delivered to smartphones during the last decade. However, there is a limit to the continued reduction of cell sizes. This limit is due to increased interference. If access points are spaced too closely together, the transmitted signals overlap (interfere) significantly. This interference enhances the noise and thus reduces the signal-to-noise ratio, sometimes to levels where it is impossible to maintain an error-free communication link. The benefit of optical wireless networking in comparison with RF wireless networking is that the limit, where interference becomes dominant, is significantly lower. This means much smaller cells can be generated while interference is kept at low levels. This is because it is much easier to spatially control light waves compared with radio waves. Microwaves, for example, pass through opaque walls. By contrast, light waves are completely blocked by opaque objects. Also, it is possible to confine the coverage by using simple lenses in an optical subsystem. Nonetheless, due to multipath propagation and random orientation of mobile devices, there is still interference present in optical wireless networks. This theme issue includes a review article on interference management in optical wireless by Little *et al.* [38], who have been at the forefront developing key techniques for light based wireless networking systems.

### (g) Networking and protocols

Lastly, the vision is that optical wireless networks will be seamlessly integrated with existing RF wireless networks [39]. This is seen as an evolutionary process since there are many different RF wireless networking technologies such as 4th generation (4G), 5th generation (5G) cellular networks as well as WiFi. Interoperability between these networks is enabled through the concept of heterogeneous networks. Optical wireless networks will be another such networking technology which is expected to be seamlessly integrated. However, this requires dynamic network management algorithms. SDN is one such dynamic network management technology which is based on the principles of network virtualization and the establishment of separate control planes and data planes. Optical wireless networks need to be designed to support these paradigms, and hence it is necessary to develop bespoke SDN agents which act as brokers (hypervisors) between specific optical wireless networking properties and general transport mechanisms and application specific requirements. These SDN agents will translate these generic requirements via the control plane into network configurations and they will cooperate to ensure that at any time the optimum connectivity to a mobile terminal is provided. For example, an optical wireless SDN agent cooperates with a WiFi agent to ensure that data flows are managed optimally. This, for example, could mean that particular users are served by a WiFi network and by an optical wireless network simultaneously by means of multipath TCP. Here the network hypervisor directs the specific data flows based on the characteristics of the respective communication links (RF and optical). Elmirghani *et al.* [40] have developed novel optical wireless networking architectures. This special issue includes a research paper on optical cloud and fog network architectures led by this group.

## 3. Summary

We are absolutely delighted to be able to attract some of the world's finest experts to contribute to this special theme issue on OWC networks. The research challenges towards the realization of optical wireless networks are multidisciplinary in nature, spanning the areas of device physics, communication system modelling, signal processing, communication theory and algorithms and computer science. This theme issue has attempted to provide a complete and comprehensive treatment of all relevant research areas across all the above disciplines. Moreover, an outlook of where the entire field might be taken is also included in this collection of papers. We hope that all active researchers in this field will get inspired by the attempt to connect related research areas which together define optical wireless networks.

## Footnotes

1 Notice this is more than the entire RF spectrum, which is 300 GHz.

## Data accessibility

This article has no additional data.
